# Laser Acupuncture at HT7 Acupoint Improves Cognitive Deficit, Neuronal Loss, Oxidative Stress, and Functions of Cholinergic and Dopaminergic Systems in Animal Model of Parkinson's Disease

**DOI:** 10.1155/2014/937601

**Published:** 2014-08-05

**Authors:** Jintanaporn Wattanathorn, Chatchada Sutalangka

**Affiliations:** ^1^Department of Physiology, Faculty of Medicine, Khon Kaen University, Khon Kaen 40002, Thailand; ^2^Integrative Complementary Alternative Medicine Research and Development Center, Khon Kaen University, Khon Kaen 40002, Thailand; ^3^Department of Physiology, Neuroscience Program, Faculty of Medicine, Khon Kaen University, Khon Kaen 40002, Thailand

## Abstract

To date, the therapeutic strategy against cognitive impairment in Parkinson's disease (PD) is still not in satisfaction level and requires novel effective intervention. Based the oxidative stress reduction and cognitive enhancement induced by laser acupuncture at HT7, the beneficial effect of laser acupuncture at HT7 against cognitive impairment in PD has been focused. In this study, we aimed to determine the effect of laser acupuncture at HT7 on memory impairment, oxidative stress status, and the functions of both cholinergic and dopaminergic systems in hippocampus of animal model of PD. Male Wistar rats, weighing 180–220 g, were induced unilateral lesion at right substantianigra by 6-OHDA and were treated with laser acupuncture continuously at a period of 14 days. The results showed that laser acupuncture at HT7 enhanced memory and neuron density in CA3 and dentate gyrus. The decreased AChE, MAO-B, and MDA together with increased GSH-Px in hippocampus of a 6-OHDA lesion rats were also observed. In conclusion, laser acupuncture at HT7 can improve neuron degeneration and memory impairment in animal model of PD partly via the decreased oxidative stress and the improved cholinergic and dopaminergic functions. More researches concerning effect of treatment duration are still required.

## 1. Introduction

Cognitive deficit, a common nonmotor feature of Parkinson's disease (PD), produces a great impact on the quality of life of patients and caregivers as well as annual healthcare cost [1–3]. It has been reported that approximate one-fifth of newly diagnosed PD patients develop the fulfilled clinical criteria for mild cognitive impairment (PD-MCI) [4] and around one-sixth develop dementia after 5 years [5].

To date, the exact mechanism of cognitive impairment in PD is still unclearly known. However, recent substantial evidence has demonstrated that cholinergic and dopaminergic systems play the crucial roles on the pathophysiology of cognitive deficit in PD [6, 7]. In addition, the disturbance of oxidative stress homeostasis in hippocampus also plays a role on this condition [6]. The current pharmacological interventions against this condition are still not in satisfaction level and the novel effective therapeutic strategy is still required.

Acupuncture has been long-term treating various disorders including neuropsychological disorders. HT7, an acupoint located at the ulnar end of the transverse crease of the writs in the depression on the radial side of the tendon flexor carpi ulnaris, has been long-term used for treating many neuropsychological impairments such as amnesia, insomnia, mania, epilepsy, and stupor. In addition, it also regulates the physical response to emotional stimuli such as anxiety, fear, and panic [8, 9]. TE5, an acupoint located at the posterior aspect of the forearm and midpoint of the interosseous space between the radius and the ulna, is also claimed for the central nervous system effect. It has been used for treating headache, stroke-related motor, and neurological and autonomic nerve problems in clinical practice [10]. However, it has been clearly demonstrated that the single acupoint stimulation only at HT7 can produce significant neuroprotective activity against the neuronal impairment and memory dysfunction induced by corticosterone, a stress hormone partly via the improved cholinergic function [11]. The stimulation acupoint can occur not only via needle stimulation but also via laser stimulation [12–18]. Therefore, various wavelengths of laser have been implemented in medicine for various purposes. It is believed that the stimuli must elicit “De Qi” which involves the stimulation and transmission of mechanical signal to connective tissue cells via mechanotransduction [19]. Since most of mechanoreceptors are located in the dermis especially at the superficial area of this layer and the skin of the rats did not contain abundant of pigment cells, the stimulation with laser with low penetration power such as blue laser at the wave length of 405 nm is enough to stimulate this group of receptor [20]. Moreover, it has been reported that the stimulation at HT7 acupoint either via manual or via laser can improve spatial memory impairment in various animal models [21, 22]. In addition, laser acupuncture at HT7 acupoint also improves neurodegeneration and cholinergic function in hippocampus [22]. Based on the these pieces of information, the beneficial effect of laser acupuncture at HT7, a noninvasive intervention, on cognitive deficit condition in Parkinson's disease has been considered. To the best of our knowledge, no scientific evidence concerning this issue is available until now. Thus, this study aimed to determine the effect of laser acupuncture at HT7 acupoint on memory impairment, oxidative stress status, and the function of both cholinergic and dopaminergic systems in 6-OHDA lesion rat, a validated animal model of Parkinson's disease.

## 2. Materials and Method

### 2.1. Animals

Young adult male Wistar rats, 8-week old, were used as experimental animals. They were obtained from National Laboratory Animal Center, Salaya, Nakorn Pathom. The weights of the animals on the first day of experiment are 180–220 grams. They were housed 6 per cage and maintained in 12 : 12 light : dark cycle and given access to food and water ad libutum. The experiments were performed to minimize animal suffering and the experimental protocols were approved by the Institutional Animal Care and Use Committee Khon Kaen University, Thailand (AEKKU 41/2554). All treatments in this study were performed once daily between 8.00 a.m., and 5.00 p.m.

### 2.2. Drugs and Chemicals

6-Hydroxydopamine hydrochloride (6-OHDA) was purchased from Sigma-Aldrich Co., USA. Sodium pentobarbital was obtained from Jagsonpal Pharmaceuticals Ltd., Haryana, India. All other chemical substances were analytical grade and purchased from Sigma Chemical Company, St. Louis, MO.

### 2.3. Experimental Protocol

All rats were randomly assigned to 4 groups of 12 animals each as follows:

 Group I Control group: rats in this group were exposed to sham operation and received no treatment (no laser treatment).
 Group II 6-OHDA: rats were induced partial lesion in substantia nigra by injecting 6-OHDA into the right substantia nigra.
 Group III 6-OHDA + sham acupoint + laser: rats received the administration of 6-OHDA into the right substantia nigra and laser acupuncture treatment at nonacupoint.
 Group IV 6-OHDA + HT7 + laser: rats received the administration of 6-OHDA into the right substantia nigra and laser acupuncture treatment at HT7 acupoint.

Rats had been treated with laser acupuncture once daily for 14 days after the administration of 6-OHDA. Then, they were assessed spatial memory using Morris water maze test at 1-day, 7-day, and 14-day periods after 6-OHDA injection. At the end of experiment, half of the rats were used for the histological study whereas the other half of the rats were used for biochemical assays including the determination of oxidative stress markers and the activities of acetylcholinesterase (AChE) and monoamine oxidase type B (MAO-B) in hippocampus. To determine the oxidative stress, AChE and MAO-B, hippocampus of each rat was isolated and determined the density of survival neurons, oxidative markers including malondialdehyde (MDA) level and the activities of superoxide dismutase (SOD), catalase (CAT), and glutathione peroxidase (GSH-Px) enzymes in hippocampus. To perform histological study, rats were transcardially perfused, prepared as a coronal section, and stained with cresyl violet.

### 2.4. Substantia Nigra Lesion

The animals were anesthetized by intraperitoneal injection of sodium pentobarbital (Jagsonpal Pharmaceuticals Ltd., Haryana, India) at dose of 60 mg*·*kg^−1^ BW. Each animal was mounted on a stereotaxic stand, the skin overlying the skull was cut to expose the skull and the coordinates for the substantia nigra par compacta (SNpc) were accurately measured (anteroposterior −0.5 mm from bregma, mediolateral 2.1 mm from midline, and dorsoventral −7.7 mm from the skull). Total 6 *μ*g of 6-OHDA was dissolved in 2 *μ*L 0.2% ascorbic acid saline [23] and was perfused into SNpc through a 30-gauge stainless needle. After the surgery, animals were allowed to recover from anesthesia and then placed in their cages.

### 2.5. Laser Acupuncture Treatment

Fifteen minutes before laser acupuncture treatment, all rats were anesthetized with sodium pentobarbital (40 mg*·*kg^−1^, i.p.) to minimize stress. Laser acupuncture treatment via HT7 acupoint was performed once daily for 14 days. The rats were treated with a laser instrument that operated with a continuous blue laser beam at wavelength of 405 nm, output power 100 mW and a spot diameter of 500 *μ*m at HT7 acupoint (the transverse crease of the wrist of the forepaw, radial to the tendon of the muscle flexor carpi ulnaris) or at 2–4 mm lateral to the HT7 acupoint for 10 minutes [24–26].

### 2.6. Determination of Spatial Memory

Spatial memory was evaluated via the Morris water maze. The water maze consists of a metal pool (170 cm in diameter × 58 cm tall) filled with tap water (25°C, 40 cm deep). The pool was divided into 4 quadrants (Northeast, Southeast, Southwest, and Northwest). The water surface was covered with nontoxic milk. The removable platform was placed below the water level at the center of one quadrant. For each animal, the location of the invisible platform was placed at the center of one quadrant and remained there throughout training. The times which animals spent to climb on the hidden platform were recorded as escape latency. In order to determine the capability of the animals to retrieve and retain information, the platform was removed 24 hr later and the rats were released into the quadrant diagonally opposite to that which contained the platform. Time spent in the region that previously contained the platform was recorded as retention time [27].

### 2.7. Histological Procedure

After the anesthesia with sodium pentobarbital (60 mg/kg BW), brains were subjected to transcardial perfusion with fixative solution containing 4% paraformaldehyde in 0.1 M phosphate buffer pH 7.3. After the perfusion, brains were removed and stored over a night in a fixative solution that used for perfusion. Then, they were infiltrated with 30% sucrose solution at 4°C. The specimens were frozen rapidly and 10 *μ*M thick sections were cut on cryostat. The selected sections were rinsed in the phosphate buffer and picked up on slides coated with 0.01% of aqueous solution of a high molecular weight poly L-lysine.

### 2.8. Morphological Analysis

Five coronal sections of each rat in each group were studied quantitatively. Neuronal counts in hippocampus were performed by eye using a 40x magnification with final field 255 *μ*m^2^. The observer was blind to the treatment at the time of analysis. Viable stained neurons were identified on the basis of a stained soma with at least two visible processes. Counts were made in five adjacent fields and the mean number extrapolated to give total number of neurons per 255 *μ*m^2^. All data are represented as number of neurons per 255 *μ*m^2^.

### 2.9. Determination of Acetylcholinesterase and Monoamine Oxidase-B Activities

The rats were divided into various groups as previously described in the experimental protocol. At the end of experiment, all rats were sacrificed. The hippocampus was isolated and prepared as a homogenate to determine the activities of AChE and MAO-B enzymes. The activities of AChE and MAO-B were determined by using the colorimetric method [28, 29].

### 2.10. Determination of Oxidative Stress Markers

Hippocampus was isolated and prepared as hippocampal homogenate and the determination of the oxidative stress markers in hippocampus was performed. Malondialdehyde (MDA) level was indirectly estimated by determining the accumulation of thiobarbituric acid reactive substances (TBARS) [30]. In order to determine the activities of antioxidant enzymes including superoxide dismutase (SOD), catalase (CAT), and glutathione peroxidase (GSH-Px), hippocampus of each rat was weighed and homogenized with a buffer consisting of 10 mM sucrose, 10 mM Tris-HCl, and 0.1 mM EDTA (pH 7.4). Then, a hippocampal homogenate was centrifuged at 3000 g at 4°C for 15 min. The supernatant was separated and used for bioassays. The activity of SOD was determined using a xanthine/xanthine oxidase system for the production of superoxide radical and subsequent measurement of cytochrome *c* as a scavenger of the radicals. Optical density was determined using a spectrometer (UV-1601, Shimadzu) at 550 nm [31]. SOD activity was presented as units per milligram of protein (U mg^−1^ protein). One unit of enzyme activity was defined as the quantity of SOD required to inhibit the reduction rate of cytochrome *c* by 50%. CAT activity in the supernatant was measured by recording the reduction rate of H_2_O_2_ absorbance at 240 nm [32]. The activity of CAT was expressed as *μ*mol H_2_O_2_ *·*min^−1 ^mg^−1^ protein. GSH-Px was determined using* t*-butyl hydroperoxide as a substrate. The optical density was spectrophotometrically recorded at 340 nm and expressed as U/mg protein [33]. One unit of the enzyme was defined as micromole (*μ*mol) of reduced nicotinamide adenine dinucleotide phosphate (NADPH) oxidized per minute.

### 2.11. Statistical Analysis

Data were expressed as means ± S.E.M. and analyzed statistically by one-way ANOVA, followed by post-hoc (LSD) test. The results were considered statistically significant at *P* value < 0.05.

## 3. Results

### 3.1. Effect of Laser Acupuncture at HT7 on Spatial Memory of 6-OHDA Lesion Rats

**Figure 1:**
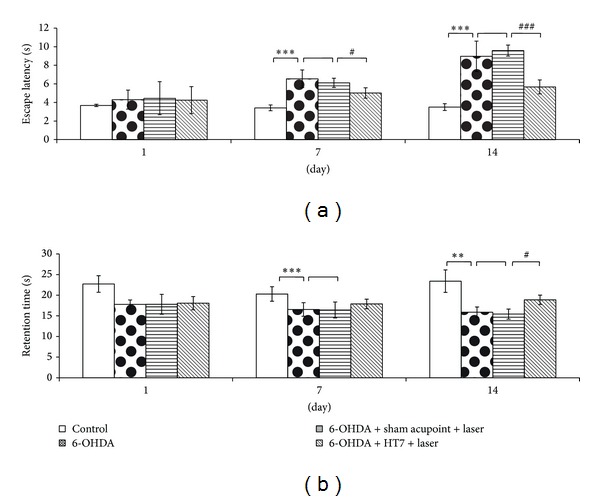
Effect of laser acupuncture on spatial memory using the Morris water maze test in Parkinson's disease rats (a) escape latency and (b) retention time. Values given are the mean ± S.D. (*n* = 6) ***P < 0.001 as compared with control group and ^#^ *P* < 0.05, ^###^ *P* < 0.001 as compared with sham laser acupuncture group.

Figures 1(a) and 1(b) showed that the administration of 6-OHDA into right substantia nigra significantly enhanced escape latency (*P* value < 0.001 all; compared to control group) but decreased retention time (*P* value < 0.001 and 0.01, respectively; compared to control group) at 7-day and 14-day period after the 6-OHDA. It was found that sham laser acupuncture failed to improve the alteration of both escape latency and retention time induced by 6-OHDA. Interestingly, laser acupuncture at HT7 acupoint significantly improved the reduction of escape latency induced by 6-OHDA both at 7-day and 14-day intervention period (*P* value < 0.05 and 0.001, respectively; compared to sham laser acupuncture group) while it showed the significant increase in retention time in 6-OHDA lesion rats only at 14-day day intervention period (*P* value < 0.05; compared to sham laser acupuncture group).

### 3.2. Effect of Laser Acupuncture at HT7 on Hippocampal Neurons

**Figure 2:**
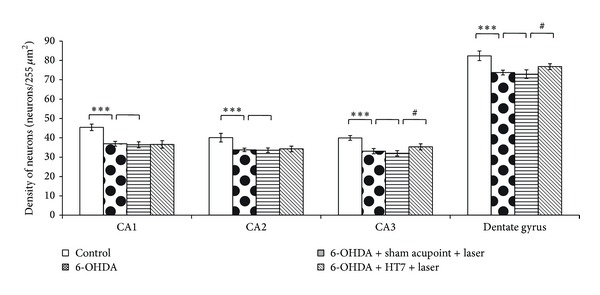
Effect of laser acupuncture on the neurons density in various subregions of hippocampus after treatments. Values given are the mean ± S.D. (*n* = 6) ***P < 0.001 as compared with control group and ^#^ *P* < 0.05 as compared with sham laser acupuncture group.

**Figure 3:**
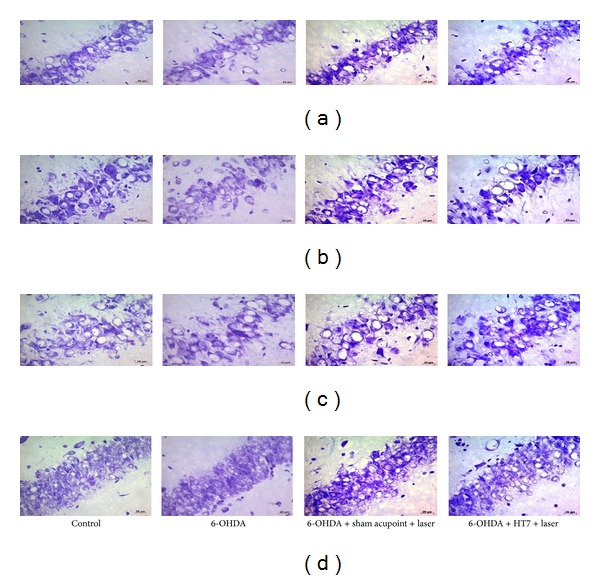
Photographic image of neurons with cresyl violet stained in various subregions of hippocampus. (a) CA1, (b) CA2, (c) CA3, and (d) dentate gyrus.

The administration of 6-OHDA into right substantia nigra induced the decreased survival neuron density in CA1, CA2, and CA3 and dentate gyrus of hippocampus (*P* value < 0.001 all; compared to control group). Sham laser acupuncture failed to mitigate the reduction of survival neuron density in all subregions of hippocampus mentioned earlier. However, laser acupuncture at HT7 acupoint significantly attenuated the decreased neuron density induced by 6-OHDA in CA3 and dentate gyrus (*P* value < 0.05 all; compared to sham laser acupuncture group) as shown in Figures 2 and 3.

### 3.3. Effect of Laser Acupuncture at HT7 on AChE and MAO-B

**Figure 4:**
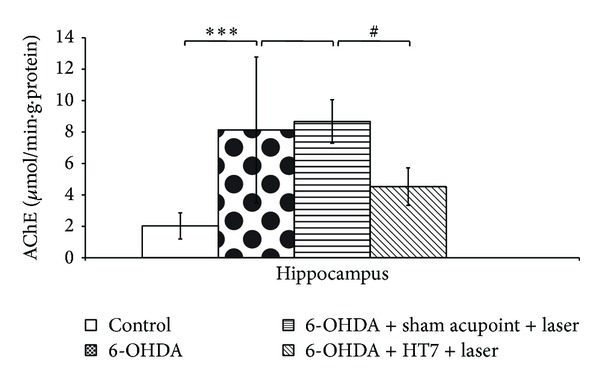
Effect of laser acupuncture on the activity of acetylcholinesterase (AChE) in the hippocampus. Values given are the mean ± S.D. (*n* = 6) ***P < 0.001 as compared with control group and ^#^ *P* < 0.05 as compared with sham laser acupuncture group.

In this study, the activity of AChE was used as indirect index to reflect the function of cholinergic whereas the activity of MAO-B was used as indirect index to reflect the function of monoaminergic especially dopaminergic system. The rats subjected to the unilateral lesion of substantia nigra induced by 6-OHDA showed the elevation of AChE in hippocampus (*P* value < 0.001; compared to control group). This change was mitigated by laser acupuncture at HT7 acupoint (*P* value < 0.05; compared to sham laser acupuncture group) while no significant change was observed in sham laser acupuncture treated group as shown in Figure 4.

**Figure 5:**
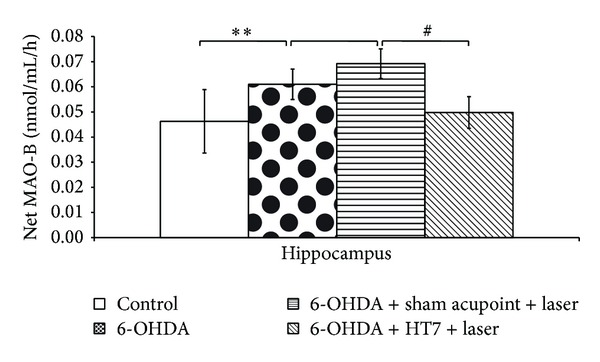
Effect of laser acupuncture on the activity of monoamine oxidase-B (MAO-B) in the hippocampus. Values given are the mean ± S.D. (*n* = 6) **P < 0.01 as compared with control group and ^#^ *P* < 0.05 as compared with sham laser acupuncture group.

Figure 5 showed the effect of laser acupuncture on MAO-B activity in hippocampus. Rats with the unilateral lesion of substantia nigra induced by 6-OHDA demonstrated the significant reduction of MAO-B in the mentioned area (*P* value < 0.001; compared to control group). Sham laser acupuncture failed to mitigate the elevation of MAO-B activity whereas laser acupuncture at HT7 significantly decreased the elevation of MAO-B activity in hippocampus (*P* value < 0.05; compared to sham laser acupuncture group).

### 3.4. Effect of Laser Acupuncture at HT7 on Oxidative Stress Markers

It was found that the administration of 6-OHDA into right substantia nigra significantly decreased CAT and GSH-Px activities but increased MDA level in hippocampus (*P* value < 0.05, 0.001 and 0.001, respectively; compared to control group). Sham laser acupuncture failed to produce significant changes of CAT and GSH-Px activities and MDA level induced by 6-OHDA in hippocampus. However, laser acupuncture at HT7 could significantly mitigate the decreased GSH-Px activity (*P* value < 0.05; compared to sham laser acupuncture group) and the elevation of MDA level (*P* value < 0.01; compared to sham laser acupuncture group) as shown in Table 1.

## 4. Discussion

The current study has clearly demonstrated that the administration of 6-OHDA into substantia nigra induced the elevation of MAO-B, AChE, and oxidative stress in hippocampus together with the enhanced spatial memory. The possible explanation for this phenomenon might be attributed to the disturbance of dopaminergic function in substantia nigra induced by 6-OHDA produced the disturbance in function of striatum via nigrostriatal pathway and striatum in turn induced the functional disturbance of hippocampus via the connection between ventral striatum and hippocampal pathway which plays a critical role on the association contextual-position information [34]. In addition, several lines of evidence have demonstrated that dentate gyrus of the hippocampus received the dopaminergic projection form substantia nigra (A9) [35, 36] and ventral tegmental area (A10), a structure nearby substantia nigra, via mesolimbic pathway. Therefore, the injected 6-OHDA might be transported into the nerve terminals in the area of both substantia nigra and ventral tegmental area and induced the disturbance of hippocampus via the mesolimbic connection mentioned earlier [6]. However, the precise underlying mechanism is still unclearly known and required further investigation.

Laser acupuncture at HT7 is demonstrated to suppress AChE in hippocampus together with the improved memory impairment in animal model of Alzheimer's disease [37]. Our findings are also in agreement with this study; the stimulation of HT7 acupoint can produce significant suppression of AChE and memory improvement even in animal model of PD. In addition to the suppression of AChE, the suppression of MAO-B in hippocampus was also observed in this study. It has been reported that laser beam could suppress MAO-B in erythrocyte of patients attacked with PD [38]. Although, laser can suppress MAO-B, sham laser acupuncture failed to suppress this enzyme activity in hippocampus. Therefore, the suppression MAO-B observed in this study might be associated with the stimulation of HT7.

In this study, the decreased MDA together with the increased GSH-Px enzyme activity induced by laser acupuncture at HT7 was observed. The discrepancy between our findings and the previous findings of Sutalangka et al. [37] which showed that no significant change of MDA was observed even the elevations of SOD and CAT were presented might be due to the different conditions of animal. Our study focused on the hypodopaminergic function induced by 6-OHDA whereas the previous study focused on the hypocholinergic condition induced by cholinotoxin, AF64A. Therefore, these data suggested that the effect of the stimulation of meridian and laser beam was varied depending on the pathological condition.

**Figure 6:**
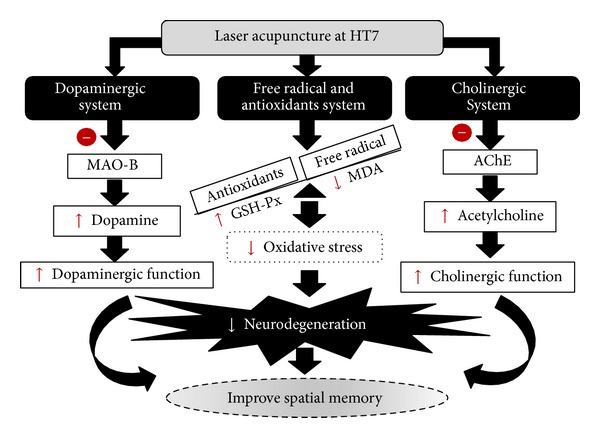
Schematic diagram illustrates the possible underlying mechanism of laser acupuncture at HT7 acupoint.

Both dopamine and cholinergic are reported to play a crucial role on memory impairment in PD [39–41]. In addition, the impairment of spatial memory is associated with the degeneration of hippocampus [42, 43] which is under the influence of the elevation of oxidative stress [35, 36]. Therefore, we did suggest that laser acupuncture at HT7 improved memory impairment in animal model of PD via the suppression of AChE and MAO-B which in turn increased the function of cholinergic and dopaminergic systems. In addition it also decreased oxidative stress via the enhanced GSH-Px enzyme activity in hippocampus giving rise to the increased oxidative stress buffering capacity resulting in the decreased neurodegeneration in the mentioned area and finally improved memory impairment as shown in Figure 6.

## 5. Conclusion

This study clearly demonstrates that the stimulation at HT7 acupoint with laser beam, a noninvasive tool, successfully improves the disturbances of neurotransmitters especially ACh and DA and oxidative stress resulting in the improved memory deficit in 6-OHDA lesion rats, a validated animal model of PD. Therefore, laser acupuncture may be a beneficial tool which is noninvasive for treating cognitive impairment in PD. The other benefit on motor symptoms of PD may also possible. However, further researches are necessary.

## Figures and Tables

**Table 1:** Effect of laser acupuncture on oxidative stress markers including MDA level and the activities of SOD, CAT, and GSH-Px enzymes.

Group	MDA (*μ*/mg protein)	SOD (*μ*/mg protein)	GSH-Px (*μ*/mg protein)	CAT (*μ*/mg protein)
Control	0.0004 ± 0.0001^###^	4.007 ± 0.334	0.387 ± 0.040^###^	140.108 ± 8.691^#^
6-OHDA	0.0011 ± 0.0003∗∗∗	3.457 ± 0.608	0.232 ± 0.057∗∗∗	114.708 ± 9.873∗
6-OHDA + sham acupoint + laser	0.0010 ± 0.0002∗∗∗	3.505 ± 0.373	0.217 ± 0.035∗∗∗	111.688 ± 8.716∗∗
6-OHDA + HT7 + laser	0.0007 ± 0.0002^∗∗##^	3.615 ± 0.453	0.297 ± 0.058^∗∗#^	132.512 ± 7.356

Values given are the mean ± S.D. (*n* = 6) *P < 0.05, **P < 0.01, and ***P < 0.001 as compared with control group and ^#^ *P* < 0.05, ^##^ *P* < 0.01, and ^###^ *P* < 0.001 as compared with sham laser acupuncture group.

## Abbreviation

PD: Parkinson's disease
6-OHDA: 6-Hydroxydopamine
SNpc: Substantia nigra par compacta
AChE: Acetylcholinesterase
MAO-B: Monoamine oxidase type B
MDA: Malondialdehyde
SOD: Superoxide dismutase
CAT: Catalase
GSH-Px: Glutathione peroxidase
*µ*: Micro
NADPH: Nicotinamide adenine dinucleotide phosphate
u: Unit
H_2_O_2_: Hydrogen peroxide
M: Mole
m: Milli
ACh: Acetylcholine
DA: Dopamine.
